# A Practical Treatise on the Diseases Peculiar to Women

**Published:** 1843-10-01

**Authors:** 


### A Practical Treatise on the Diseases peculiar to Women. By *Samuel Ashwell,* M.D. Part II. pp. 250, 8vo. 1843.

This is a continuation of the Work we had occasion to speak very favorably of in our Number for January 1841. It completes the subject of functional and commences that of organic diseases of the internal genitals. A part to be published soon will complete the latter, and treat of the affections of the external parts. The analysis we propose to give demands all our space.

### Hysteria.

Hysteria Dr. Ashwell considers " essentially to consist in excitement and irritation of the numerous and important nerves supplying the reproductive system." There are few practitioners but have been deceived by this proteiform malady into the belief of the existence of important affections, and few who cannot call to mind the satisfaction they have experienced in being able to establish that certain alarming symptoms arose from the hysteric diathesis. Quite aware of this, the author yet impresses upon us that we must always remember that hysteria itself has its limits, and that not only may congestive and inflammatory diseases be mistaken for it, but that it may itself terminate in these. No one can doubt that many local affections have been too hastily classed as hysterical, and treated empirically, but we feel certain, that the prevalent error of young practitioners is to run into the opposite extreme, and treat every painful affection as if of an undoubted inflammatory nature. It is consoling to remember that " where the hysteric diathesis really prevails, recoveries sometimes occur from states in which all hope has been laid aside. Thus paralysis, and difficulty of swallowing, and great debility, are extraordinarily recovered from; and, occasionally, when phthisis and the emaciation supposed to be its direct result, have reached an apparently hopeless point, the patient most singularly and inexplicably recovers." The author has also observed that diseases destructive of life, especially phthisis, are slower in their progress in the hysterical.

The *diagnosis* of a disease like hysteria, which simulates so accurately so many other affections, is both difficult and important, and is chiefly accomplished by the observance of incongruities in the assemblage or sequence of certain sets of symptoms, which, in the disease with which it is confounded, are found to be consentaneous. Even the paroxysm itself, although usually easily distinguished, may be sometimes mistaken for that of epilepsy. Marshall Hall observes that, much as the larynx may be affected in hysteria, it remains unclosed, and we have heaving, sighing inspiration, while, in epilepsy, we find violent, ineffectual efforts at expiration. The epileptic seizure is usually more sudden, and the deprivation of consciousness more complete; and it occurs more frequently in men than women. There is seldom biting of the tongue, or frothing of the mouth, in hysteria, nor is it followed by the heavy sleep, and insensible pupil. The physiognomy of one liable to epilepsy soon becomes peculiar.

*Treatment.*—The author truly observes that few practitioners like to be saddled with the treatment of a case of true hysteria; but he recommends those who do undertake it to thoroughly investigate the condition of the general health and of the various functions, instead of merely attacking the local symptoms which may present themselves, and which may lead him a chace over the entire frame. It too often happens that, however judiciously the treatment may be conducted, it proves of little avail. During the *paroxysm*, generally, little need be done beyond the application of cold water and a smelling-bottle. "It has already been observed, that consciousness is generally retained, and enough of volition, except in convulsive and epileptic hysteria, to enable the individual to avoid danger; so that as the fit, by equalizing the circulation, and by removing nervous irritation, has upon the whole a beneficial effect, restraint need form but a small part of the treatment. A lady, whom I long attended, always rejoiced when the fit was over, because it relieved her system generally, and especially her brain, from painful irritation, which had often existed for several previous days." Encouragement to use voluntary efforts to repress the paroxysm, cautiously practising on the fears of the patient, or the swallowing of iced water, have each been found useful. When there is no plethora diffusible and fetid stimuli may be given; but, where plethora exists, and important discharges are suppressed, a moderate bleeding, or rather cupping low down between the shoulders, is of service. When there is great rigidity of the muscles of the head and trunk, and impaired deglutition, turpentine, or cold water glysters should be administered. The mustard bath, as high as the knees, is a good derivative, and "on some occasions I have known the attack quickly terminated by ringing a loud and shrill-sounding bell close to the ear for several minutes." An approaching attack may sometimes be warded off by the affusion of cold water, its injection into the rectum, or the swallowing half a drachm of ipecacuanha.

*The Treatment in the Interval,*—will require modification according to several circumstances. A *morbid condition of the uterine system* is very frequently found in hysteria, as is seen, in girls who menstruate with difficulty, ill-assorted marriages, young widows, &c.

"I am aware it may be urged, in opposition to these opinions, that structural lesions of the uterus are very common in women who have never had hysteria. Of the truth of this statement, to its full extent, I am more than doubtful: as I have accurately ascertained, both in hospital and private practice, that such individuals are by no means, especially in early life, so exempt from this common malady: nor must it be forgotten, although there are exceptions, that these affections generally do not occur until the reproductive faculty is either about to cease naturally, or has become seriously impaired by the progress of these organic changes." 230.

Although *plethora* is not a common accompaniment, yet it is occasionally found, when there is defective menstruation, slothful habits, suppressed evacuations, &c.; and the usual remedies for this condition may avert the attacks, and if persevered in, in a modified manner, remove the disposition to them. Agreeing with the author in these views, we may observe, that the plethora is often rather apparent than real, and the existence of hysteria affords a *prima-facie* probability that depletory measures, to any extent, or for long continuance, will not be borne. The case of *hysteria with debility* is, Dr. Ashwell observes, far more common, and when this is, as is often the case, complicated with some local vascular congestion or excitation, it requires delicate treatment. A case in which local depletion and counter-irritation are to be combined with a good diet and tonics. The *gastro-intestinal disorder,* often attendant upon hysteria, may produce great tenderness in the hypogastric region, relievable by leeches applied to the abdomen or to the anus, or excessive flatulence, frequently itself inducing a paroxysm. This last is to be thus treated—" a small tumbler of water, as hot as it can be swallowed, during or immediately after the meal, with some powdered ginger, a little brandy, sal volatile, or a few grains of Cayenne pepper entirely dissolved in it, seldom fails to afford relief: friction by the hand or flesh-brush over the abdomen, and in really severe cases, the injection of a pint of hot water into the rectum, with or without assafœtida, may be tried."

The *diet* must be simple, nutritious and slightly stimulating, and the irritation of worms, or fæcal accumulations guarded against by the use of purgatives.

*Treatment of peculiar Symptoms.*—Notwithstanding the general treatment employed these may have to be met empirically. *Headache,* and its treatment, have already been considered in the former part of the work. In this distressing and obstinate malady the author has often found the following draught useful. ℞. Tr. valer., amm., sp. eth. s. c., Sp. lav. c. āā ʒss., Tr. hyoscy. ♏xx. M. camph. ℥x. 2da vel 3tia horâ. *Pain of the left side* is perhaps the most difficult of all hysterical symptoms to relieve. The author does not agree with Mr. Tate, in referring this generally to spinal affection, and has seen much harm result, both from the rough manipulations employed to ascertain whether tenderness along the column existed, and from the depletion and counter-irritation employed to relieve it. The whole armoury of the Pharmacopœia has frequently been exhausted in vain in these cases. Dr. Ashwell has found the following liniment useful. ℞. Ether. rect., sp. camph., tr. opii., tr. lyttæ āā ʒiv. M.

In regard to hysterical females acting as *nurses* the author observes:

"It is not unimportant to observe, that although marriage often cures hysteria, women who have *long suffered* from its effects, rarely make good nurses. Doubtless there are exceptions to this fact; nor is it intended to be urged, that such women cannot suckle at all; nor that they may not occasionally be benefited by lactation. But where, prior to a late marriage, hysteria has existed for years, in association with extreme susceptibility, peevishness of disposition, and thinness of person, it is for the most part undesirable that such mothers should suckle their offspring. The milk is often disordered, the child's digestive system is thereby deranged, and a predisposition to nervous disease may be communicated." 258.

### OF THE IRRITABLE UTERUS OR HYSTERALGIA.

"DEFINITION.—*A permanent and painful sensibility of the uterus, and especially of its neck: often accompanied by increased frequency of pulse, a dry hot skin, and generally, in protracted cases, with stomach and renal derangement. The disease usually occurs during the middle period of life, and commonly prevents conception. It is exceedingly difficult to cure, even to palliate: and, it is said, that it is neither attended by, nor tends to produce change of structure.*

This disease, Dr. Ashwell remarks, both as to symptoms and treatment required, resembles a permanent dysmenorrhœa. He has never met with it at an earlier age than 23, but Dewees has seen it at 18. It occurs only in the married. The severe pain and suffering characterizing the malady are much exasperated by the slightest movement or exertion, and especially by the motion imparted to the cervix, during the efforts at stool, or in passing urine. Excessive agony is also produced if the cervix be rudely touched during an examination: but Dr. Ashwell does not confirm the statement of Dr. Gooch, that the tenderness is confined to the cervix, and does not extend to the vagina. The contrary is the case, although such limitation exists in the rarer disease of acute inflammation of the cervix. The uterus is found low down in the cavity of the pelvis, or even prolapsed, and its cervix is often somewhat shortened, and expanded, and occasionally puffy and swollen, and the lips of the os are more than naturally closed. The suffering is greatest about the period of menstruation, which continues regular, but scanty. In three of the author's cases, the disease has gradually disappeared with the decline of the catamenia, without leaving any morbid condition of the cervix. He does not, however, agree with Gooch, that this is a mere state of irritation, but believes, that examination by the speculum, and the effects of remedies, both prove a condition of subacute inflammation to exist, and that, in some cases, this may go on to structural change. The predisposing causes are of difficult discovery, for, although the disease chiefly occurs in those of a highly nervous temperament, it may do so in the robust. Life is seldom in danger, but the case is always tedious, and even the degree of relief to be obtained, and freedom from frequency of relapse, will much depend upon the perseverance and quietude of the patient. The treatment is very much like that already recommended by the author for dysmenorrhœa. Repose is essential, perhaps for months; active purging is injurious: a generous yet non-stimulant diet is requisite: steel in moderate doses and long-continued, and narcotics in small quantities, and frequently varied, are the best medicines. Low diet, drastics, close rooms, and general bleeding are injurious. In several cases Dr. Ashwell has found the scarification of the cervix, as recommended by Mr. Fenner, or applications of leeches to that part, very beneficial. This is only the case when there is evidence of encreased congestion and vascularity, which indeed are usually present; but in simple neuralgia, when the cervix is even small and shrivelled, change of air, sea voyage, chalybeates, and means of that description, are to be employed. Pessaries, even where no marked uterine descent exists, provided the vagina be not too tender and irritable, will give great relief. "In several instances every other measure was fruitlessly employed: but the use of a circular box-wood pessary, for three or four months, seemed really to have cured the affection."

### General Remarks on the Organic Diseases of the Uterine System.

In investigating organic diseases of the uterus we avail ourselves of several means of inquiry, such as the history of the symptoms, the touch, speculum, stethoscope, and inspection of the discharges. The degree of *Pain* present in the early stages is frequently so slight, as to prevent attention being drawn to the affections until too late to remedy them, while, in some cases, even the latter stages are attended with very little suffering. In functional and inflammatory disorders acute pain early manifests itself. Even when pain does occur it is not characteristic as regards each affection, but, arising chiefly from increased bulk and the displacement of other organs, it is common to several.

*Emaciation* is almost always present, in malignant disease, but in hard tumors of the uterus it frequently does not occur until the latter stage. The *examination by the hand* is most important, not only to detect what description of organic disease may be present, but also whether this be complicated with pregnancy: "for, if there be no fœtus in utero, a palliative treatment will be proper, whereas, if the patient be pregnant, her safety mainly and almost solely depends on the induction of premature labour." In regard to the mode of making the examination.

"The patient must be placed on her left side, the usual obstetric position; and the labia and nymphæ being carefully separated, the forefinger of the right hand will commonly reach and touch the parts satisfactorily. It must, however, be remembered, that the sensitive part of this finger can only examine with nicety that portion of the neck and os lying opposite to it. To examine the whole circumference of the neck, the index finger of the left hand must also be used; and then it is scarcely possible that any morbid spot, or induration, can escape detection. As in the operation of lithotomy, a deep perineum increases the difficulty, so in the internal examination, an unusually long vagina, a broad perineum, and large and fat labia, present obstacles to the investigation by a single finger. In such patients, two fingers, or perhaps the whole hand, must be used —having been previously lubricated by oil, rather than by any unctuous substance." 264.

Provided we can assure our diagnosis the fewer means we make use of the better, for, while in almost every case, the touch may be employed at least once, the speculum sometimes give rise to injury of both the unhealthy parts, and the sound vagina, into which it is introduced. It is very important to remember that there may exist varieties in the cervix as to size and shape, quite independently of disease, for many, by reason of their not knowing these anatomical varieties, have supposed disease to exist which has not been present. A considerable elongation is frequently found, and women, even who have borne children, may have the cervix compact and small, and perforated by a small circular aperture instead of the normal os. Moreover, Dr. Ashwell states,

"*A large uterus, especially at its lower part, a large and soft cervix, a patulous os, fissured, indurated, and cicatrized, may all exist, without organic, and especially without active organic disease.* Prior to, during, and even soon after the catamenial flow, the body, and particularly the neck of the uterus, is larger, and more supple than natural; and imparts to the finger a similar sensation to that communicated in the early months of gestation. Frequent sexual intercourse will also induce this state of parts. During natural and healthy menstruation, the orifice of the neck is very dilatable, and easily allows the passage of the finger: this will but rarely occur at other times, independently of disease; and the opinion will be unfavourable, if the finger, on passing into the canal of the cervix, shall touch a puckered, coarse, and rough membrane. *Induration* and *cicatrisation,* in slight degree, may result from lacerations during labour, and from the inflammation attendant on their union. In old women, it is especially important to remember, that the cervix naturally diminishes in size, and the contraction of its structure is almost invariably associated with considerable induration; but still, without disease. It will not be difficult to appreciate morbid changes in the consistency of the neck: for although the cervix possesses the firmness of a gland, this may, by a practised examiner, be easily distinguished from the induration, with tenderness, of chronic inflammation: and still more easily from the almost stony or marble hardness of a scirrhous tumor. I cannot forbear to caution the practitioner against a hasty and alarming prognosis, where *unhealthy softness is connected with losses of blood and irregular catamenial discharges.* Such a condition is curable; and occasionally, where little has been done, it has continued for years, perhaps till the final departure of the catamenia; and the cervix has then acquired its usual hardness. Pain and heat, (absent when the os uteri is in a healthy condition) in high degree, are both present in inflammation of the cervix; while in the early and more advanced stages of organic disease they are often, if not generally, absent." 269.

*The Speculum.*—By gently overcoming the resistance of the sphincter vaginæ, and avoiding stretching the fourchette, the instrument may easily be introduced, but some care is then required in availing ourselves of it.

"The position of the neck is occasionally changed, being placed more forward or posteriorly than natural. To obviate this difficulty, and to bring the cervix within the end of the tube, the speculum must be elevated or depressed. Sometimes from spasmodic contraction, induced by the passing of the cylinder, a fold of the mucous membrane of the vagina is forced into the aperture of the speculum, and may be mistaken for the cervix: the least movement, however, of the instrument, will cause the slipping away of the portion thus placed; and the recognition of the neck, which is glandular, smooth, and without rugæ, and paler than the vagina, is not difficult. The whole circumference of a very large cervix cannot be examined at once: the position of the speculum requires attention; and if the parts are not morbidly sensitive, the instrument is easily and safely turned in the vagina: this caution is important, as very lately I overlooked a rather large ulcer on the inferior and posterior surface of the cervix, from a neglect of it." 271.

The appearance of the cervix is thus described:

"In health the cervix uteri is, externally, of pale colour, having the aspect of polished skin; and it is easily distinguished from the lining membrane of the vagina, which, from its different structure, and greater supply of blood, has a much deeper tint of red. These parts are naturally covered with a thick mucus; a fact of importance, as, if it be not removed by lint or a soft brush, abrasions or ulcerations, being thus obscured, might be overlooked."

The author looks upon the speculum as a most valuable instrument both for the detection of the diseases of the cervix, and for the application of the appropriate remedies, and regrets its application is sometimes delayed so long. In slight cases of leucorrhœa and uterine irritation it is prejudicial. In the very young and very old its introduction is often difficult and dangerous, and therefore not to be attempted. When the cervix is much inflamed, or the vagina very irritable, these conditions must be removed before employing the speculum.

*The Stethoscope* is of value in aiding in the detection of pregnancy, complicating organic disease of the uterus. But the placental souffle may be exactly imitated by the pressure of a tumor upon any of the large abdominal vessels.

*The Discharges.*—Much information, which can be relied upon, cannot be obtained from these independently of an examination.

"It may with truth be affirmed, that until an examination has been allowed a serous discharge has often been thought to be the proof of a malignant disease of the os, when it has really only been leucorrhœal; and a discharge of pus, mixed with blood, and slightly odorous, has equally often excited painful anxiety, lest structural disease existed, when in fact neither the finger nor the speculum could detect any mischief."

*Prognosis.*—The author truly observes, that the practitioner often finds himself in the dilemma, of delivering such a prognosis as will effectually cast down those energies of the patient, on which he relies for assistance in delaying the progress of the disease, or of risking his own reputation, by holding out hopes which the case does not warrant. He especially cautions against a too hasty and unfavorable prognosis, which the difficulty of diagnosis of some of the affections, and the increased chance of curing others, in their early stage, his investigations hold out, should alike forbid.

"Occasionally, when a quickly fatal issue has been predicted, marked relief or at least many years of life, not without comfort, and sometimes even of enjoyment, have falsified the too unfavorable and hasty opinion.

"Doubtless if there be predisposition to adventitious heterologous formations, such causes will favour their development; but if there be no such constitutional tendency, these unhealthy states may continue long without assuming an incurable character. In women, the mothers of numerous families, I have several times, independently of any change in the body of the organ, found the cervix large and hard; and when treatment had been long laid aside, I have, years afterwards, ascertained that although these conditions continued, yet that there was no development of malignancy." 279.

### The Tumors of the Walls of the Uterus characterized by Induration.

These tumors of a fibrous or even calcareous hardness present every variety as to size. They frequently do not excite attention until by their magnitude they cause pelvic obstruction. The author considers them as a scirrhous variety of carcinoma. They present different symptoms accordingly as they arise from the external or internal portion of the uterus. In reference to those growing *externally* it may be observed, that, sometimes after reaching a certain magnitude, they may remain stationary for years, while, in other cases, after a period of quiescence, their progress becomes inordinately rapid. They do not prevent conception, and this indeed sometimes happens when they have existed for years, and when the period for its occurrence might be supposed to have passed. Notwithstanding the author entertains a strong opinion that these growths are of a cancerous nature, he recommends their treatment by means of iodine.

"The inferences I have drawn from the use of this medicine are as follow: 1st. Its internal administration, and its use, by inunction, is decidedly beneficial; the advantage, if the remedy be judiciously employed, being rarely attended by constitutional injury. 2nd. In hard tumors of the walls, or cavity of the uterus, resolution or disappearance is scarcely to be expected; since the growths are adventitious or parasitic, and are not imbedded in glandular structure. Here the prevention of farther deposit—in other words, the restraint of the lesion within its present limits, and the improvement of the general health—will be the extent of the benefit derived. It must not be supposed that the use of iodine is empirically to preclude the employment of other means: cupping on the loins; a mild, animal, unstimulating, and often, for a time, a milk diet; gentle aperients, and the warm poppy hip-bath, are important adjuvants. In the appended cases it will be seen that I have employed leeches and setons, with marked advantage; and there can be no doubt that sexual excitement must often exercise a prejudicial influence." 293.

The *Submucous Variety*.—Although of less size and induration, yet projecting towards the uterine cavity, and covered with its mucous membrane, these give rise to more dangerous consequences than the tumors already mentioned. It is well known that they rarely ulcerate, unless they occupy the neck or mouth of the womb; but, independently of ulceration, they cause alarming symptoms by the violent hæmorrhages they induce—the blood proceeding, not from the substance of the tumor itself, but from the mucous membrane covering it. It is not common for the tumors to encroach much on the cavity of the uterus. Their indurated structure, traversed with white lines, the circumstance of their being sometimes numerous, and the thickening and induration of the uterus near their site, distinguish them from *polypi*, into which they are never, as imagined by some, converted. The polypus is vascular and easily injected, but not sensible, while the contrary is the case with the hard tumor. Pregnancy may co-exist with the tumor, but rarely with polypus. A ligature which would remove a polypus, would probably include a portion of the contiguous uterine structure, if applied to the tumor. The dangerous hæmorrhages so frequent in these cases, render the adoption of palliative means even, and which is indeed all they admit of, of great consequence. Rigid abstinence from sexual intercourse, the recumbent posture, and a moderate antiphlogistic treatment are required. Narcotic suppositories, and anodyne vaginal injections are useful. Iodine may be employed, but the author possesses no evidence of its utility. By such means, when the disease is discovered sufficiently early, life may usually be prolonged, the progress of the tumor may be arrested for a time, and the congested state of the uterus become diminished. Mere palliation, and partial exemption from the hæmorrhage form usually the extent of the benefits conferred.

### On the Induction of Premature Labour in Pregnancy complicated with Organic Diseases.

The propriety of this practice the author considers to be established by the two following propositions.

"1st. That when death occurs, after a labour so complicated, the result is only slightly, if at all, referible to the uterus, which rarely sustains any mischief; but is mainly produced by inflammation, softening, and unhealthy suppuration in the growth itself: these pathological changes leading, in some instances, to rapid sinking; while in others, the powers of the system having been less impaired, death ensues in a few days, from the constitutional collapse, induced by the protraction and difficulty of parturition, and by the contusion and injury done to the tumor and other soft parts. And 2ndly. That premature parturition, artificially induced, rarely occasions constitutional mischief; is easily accomplished, and affords the best, and, in many instances, the only chance of a safe result to the mother." 323.

In reference to the *first* of these, he observes, that tumors obstructing parturition give rise to some of the most serious of obstetric difficulties, for the extent of the obstruction cannot be ascertained with the same facility as when such arises from a contracted pelvis: and even, when the mere difficulties of parturition have been overcome by instrumental assistance, instead of the woman quickly recovering, as she usually does after ordinary instrumental labours, a new train of symptoms, originating in the violence which has been done to the tumor, become developed. In all the cases Dr. Ashwell has seen, and in most he has perused accounts of, the uterus itself, in patients dying under these circumstances, has been found unaffected. If these observations hold good in reference to tumors, characterized by mere bulk and induration, they do so in a yet greater degree when a malignant character of the growth prevails. The best practice, prior to the discovery of inducing premature labour, consisted in puncturing and evacuating, as far as possible, the contents of the tumor, and thus relieving it from the injurious and destructive pressure alluded to.

As to the *second* proposition, the author's attention was first drawn to the operation by his having, in one or two cases of labour, complicating tumors of the uterus, during his examinations, separated accidentally the membranes to some extent from the os uteri—a happy accident, indeed, for premature labour coming on, the patients escaped the ill consequences, which would have attended, had they gone to their full period. Dr. Ashwell has no doubt as to the complete safety of the practice. He has induced premature labour in a great number of cases, and has rarely found the operation followed by sufficient excitement to give rise to the least anxiety.

He farther adds—

"Fortunately, pregnancy complicated with tumor, is not of such common occurrence as pregnancy with a narrow and deformed pelvis; and, consequently, the necessity for the intervention of art has not been so urgently pressed upon the attention of professional men. Still, if the contingency is more rare, when it does arise, it is infinitely more dangerous, at least if the tumor be large, and so situated as to obstruct parturition. Seeing, then, how admirably the dangers of the former class of difficult labours are evaded by premature parturition, I may be allowed to express surprise that this operation has not been resorted to in these more formidable complications. I contend, therefore, that this practice is peculiarly applicable to these cases, almost independently of any reference to the life of the child. In support of this opinion, it may be observed, that there is a vast difference between cases of pelvic narrowing and deformity, where there is nothing to preclude the safety and desirability of pregnancy but the faulty conformation, and cases of morbid growths and diseased enlargements of organs, which render it urgently important, that impregnation should not occur; or, if it have occurred, that it should not so far advance, as to call into activity the dormant energy of the tumor. The life of a fœtus is, under such circumstances, of comparatively little moment.

"Another point of importance is, the time when premature labour should be brought on. A variety of circumstances will influence the decision of this question. If the practitioner enjoys the advantage of an early introduction to his patient; if he possess tact enough nicely to examine the bulk and attachments of the tumor; and if he has accurately noted the first attacks of pain in it, and the constitutional impression produced by them; he will not hesitate much as to the precise period when to adopt this measure. I should not delay, if there were constant pain in or near the growth; if the respiration were embarrassed; and if, as a consequence of these conditions, the pulse were quick and irritable, the extremities œdematous, and the functions of the kidneys and the skin were partially or greatly interrupted. When such an amount of evil exists, or rather before the entire series of these symptoms is complete, the moment has arrived to empty the uterus; and I venture this opinion with the more confidence, conceiving that pregnancy will rarely give rise to this amount of mischief till the 6th, or perhaps nearly the 7th, month of gestation, when, if turning shall be required, it may be accomplished with only the usual difficulties and hazards." 329-33.

The author cites the practice and opinions of Dr. Robert Lee, as confirmatory of this practice, and of extending the category of cases to which it is applicable, *e, g.* cancer and excrescences of the cervix, malignant disease of the external genitals, and whenever ovarian disease, ascites, heart affections, obstinate vomiting, &c. threaten life.

### CONGESTION AND INFLAMMATION OF THE UTERUS.

Before proceeding to treat of the carcinomatous condition of the os uteri, Dr. Ashwell offers some remarks upon enlargement and induration of this part, independently of malignancy, and arising from a congested or inflamed condition of the uterus.

*Congestion.*—The author does not agree with those who look upon the state of congestion of the generative organs at the menstrual periods, as attended with serious evil, which, however, may arise, when it is protracted, or terminates in actual inflammation. He considers the fact of the continued regularity of the catamenia, as one principal reason why so many of the organic diseases of the uterus are of such slow growth—many of them, indeed, only appearing after this has ceased, when any increased determination of blood, unrelieved by the menstrual discharge, becomes stationary and mischievous. Congestion may also arise in amenorrhœa, when the progress of the uterine disease will be accelerated, and its severity increased—to be again retarded on the regular re-production of the menstrual flux. It may also be induced by various exciting causes, as excessive venery, mental emotion, inordinate horse or foot exercise, frequent abortion, &c. When the fulness and uneasiness about the pelvic viscera, accompanied by occasional hæmorrhage, has attracted attention, and an examination is made, the uterus is usually found prolapsed, giving to the finger a swollen, œdematous, or doughy feeling, and having its os spongy and patulous. It is rare to find tenderness or heat, but the speculum shows a shining, injected, and venous colour, and frequently a slight exudation. When it occurs merely at the menstrual periods medical treatment is seldom sought, but this becomes of importance, when the congestion, as it usually does, complicates the various other uterine affections. Rest in the recumbent posture is essential, and sometimes sufficient, for its relief; and when it is not so, leeching or scarifying the cervix will suffice. When this is objected to, a few ounces of blood may be taken from the arm, at, or just before, the menstrual periods—applying a blister to the sacrum, in the interval. When the affection becomes chronic, the use of an alum hip-bath, formed by the addition of eight or ten ounces of alum to the water, and maintaining its temperature at from 96° or 98° for half an hour, is useful. In Summer, or when the efficacy of the remedy is to be maintained, the temperature must be diminished.

*Acute Metritis.*—This is rare in the unimpregnated state, and the parenchyma of the organ is its usual seat. The married are chiefly, but not exclusively, prone to it, and, in all, it is especially liable to occur about the period of the catamenial decline. Very severe pain, deep behind the pubis, is a prominent symptom, which is much aggravated by the movement impressed upon the cervix in the evacuation of the bladder and rectum. The treatment is obvious as soon as the nature of the disease can be recognized.

"*Chronic Metritis* is an exceedingly frequent disease, often affecting the whole organ, but more commonly confined to the cervix. Sometimes it follows active inflammation, but more usually it comes on slowly, and independently of an acute attack. There is nothing in which knowledge and accuracy are more necessary, than in the diagnosis of the conditions, and especially of the induration induced by this insidious affection. It may be regarded as the neutral ground of organic uterine disease. To know that the alterations in the cervix are still of a simple kind, after inflammation protracted through many months, or even a longer period, to feel certain that a favourable prognosis may be justly given, require close and extended observation. But I am certain that, for a much longer period than is generally supposed, a cure may be fully anticipated; and every day's experience convinces me, that assiduous treatment would accomplish far more than many practitioners venture even to hope, much less confidently to expect." * * * * *

"Still, there can be no doubt that change of organization takes place and that conversion into real uterine scirrhus is the occasional result of insidious chronic inflammation. Such a fact should be an incentive to watchful and persevering treatment." 366.

The affection is accompanied by much pelvic uneasiness and discharges of varying appearance.

"It is after the persistence of these symptoms for some weeks or months, that the sallow countenance, the impaired appetite and digestion, abdominal pain, slight emaciation, and a gradual loss of strength, excite apprehension, and an examination being solicited, is generally readily granted. The uterus is almost invariably enlarged, and often considerably indurated, and, on balancing it on the finger, its increased weight is evident. The cervix, from infiltration of lymph, is frequently hypertrophied; its mucous follicles, being filled with fibrous effusion, are prominent, and project unduly beyond the surface.

"This peculiarly elevated state of the uterine mucous follicles, has been denominated the *granular* inflammation of the cervix, a term also used, when the mucous surface of the neck is studded with the effused lymph, in the form of red or highly-coloured granulations, instead of its being infiltrated into the follicles or crypts. The cervix is more bulky and doughy in feel, and the os is found to be softer, more widely open than natural, and often, in some part of the aperture, there is tenderness or pain, with a roughness amounting almost to abrasion." 367.

In the *treatment* of this affection, topical depletion, the hip-bath, mild laxatives, entire rest, pure air, and a regulated diet, are the means called for. Iodine, mercury, conium, &c. will exert no beneficial effect in diminishing the induration and enlargement until the existing inflammation has by such a plan of treatment become alleviated. The effect of depletion in increasing the power of the absorbents, by removing existing inflammation, is known to every practitioner.

### CANCER OF THE WOMB.

1st. *The Incipient Stage.*—The author commences this chapter with the all-important question—"*Whether prevention of further mischief, presuming the disease to be in its incipient stage, or a cure of that which already exists, may be reasonably hoped for?*" It is cheering to find that a still farther extended experience, since he published his paper in the Guy's Hospital Reports, 1836, leads him to reply to this in the affirmative, while the subsequently published researches of Duparcque and Montgomery, tend to the corroboration of the statement. The passage which he quotes from the Reports contains the pith of his conclusions.

"'That hard tumors of the cervix, and indurated puckerings of the os, (conditions which frequently terminate in ulceration) may be melted down and cured by the topical application of iodine, aided by the recumbent posture, abstinence from sexual intercourse, cupping on the loins, a mild, unstimulating, and often a milk diet, gentle aperients, narcotic injections into the vagina, and the almost daily use of the warm hip-bath.'

"It has been doubted whether I have sufficiently defined the nature of these hard tumors; whether, in fact, they are to be regarded as cancerous, or merely as congestions and ulcerations, which, not being malignant, are capable of cure. I believed, at the time I wrote these observations, and I still adhere to the opinion, that they were malignant tumors; but that their full development was prevented, at this early period, by the treatment pursued; for I have long been convinced, that cancer of the womb may be arrested, in its early stages, by the removal of the pathological state, of which it is the consequence." 370.

The author, in corroboration of the possibility of relieving the early stage of cancer, quotes from the admirable paper by Dr. Montgomery, which appeared in the Dublin Journal, January 1842, and extracts from which were given in the Medico-Chirurgical Review, for the following quarter. Such cases are not to be confounded with others, in which the cancer may become quiescent for a time, still retaining all its malignancy, yet to be developed.

"I am aware it may be urged against the reality of the cure, or the arrest of the malady; that the incipient stage of cancer is occasionally protracted to several years, even where treatment is entirely neglected; but this can scarcely impugn the value of the measures now urged, as during any portion of the time the disease is thus inactive and stationary, it remains without diminution. But it is not so where powerful and persevering treatment is in efficient operation; in such cases, the disease is retrograding; the congestion, puckering, and induration, and the morbid state of the mucous lining are gradually and perceptibly lessening, facts satisfactorily proved by repeated examinations with the finger and with the speculum." 375.

The progress of cancer is very diversified, in all its stages, in different subjects. The author has observed it to be slowest in dark complexions, and most rapid in the fair and ruddy, whose capillary circulation is most active. Severe illness of any kind, or mental distress, frequently accelerates it.

*Early Symptoms.*—These, in connexion with the opinion of the curability of the early stages, are of the last importance. Pains, often of a severe character, about the back and pubes, and sometimes in the course of the sciatic nerve, at first transient, become persistent, and attract forcibly the patient's notice. Irregular menstruation, and vaginal discharges are not usually found in the early stages, but pain in sexual intercourse is so sometimes. Accurate knowledge is alone to be obtained by an examination. Three kinds of induration may be found.

"1. The rima, or circumference of the uterine aperture, may be wholly, or only partially, hardened and puckered.

"2. The cervix may be hard throughout its whole structure.

"3. Hard tumors may be deposited in any part of it.

"I am quite aware that tact, and a somewhat extensive knowledge of the normal or healthy varieties of these parts, are necessary for accurate diagnosis. The practitioner, therefore, will do well to remember, that, independently of disease, there may be—1. A large and firm cervix. 2. A capacious, patulous, and firm os. 3. An os fissured, and unequally hard." 377.

*Predisposing Causes.*—The disease is frequently hereditary. Although not often a disease of the young, Dr. Ashwell has seen it below 20, Carmichael at 21; Wigaud at 14. Boivin and Duges, out of 409 cases, found it existing, in 12 under 20; in 83 between 30 and 40; in 106 between 40 and 45; and in 95 between 45 and 50. From 30 to 55 are however the years when women are especially liable. Married women are more prone than single, or even than widows, and distress of mind would seem to exert a great predisposing effect.

*Exciting Causes,* such as blows, falls, undue pressure, violence done in parturition, or sexual intercourse, &c., may undoubtedly induce the disease, by bringing a pre-existing disposition into activity; but while in some cases these causes occurring in an aggravated degree have yet produced mere simple induration, cases of confirmed cancer come on at other times, unpreceded by any of them.

*Pathology.*—Respecting the hypothesis of the cancerous vitiation of the blood, advocated by so many able pathologists, the author observes:—

"There is no doubt that carcinomatous and encephaloid matter has been found in the interior both of veins and arteries. I cannot, however, after a careful perusal of all the reported cases of this kind, discover any proofs that these cancerous products existed in the blood or its vessels alone; in other words, independently of, or previously to, the development of the disease in the tissue of the solids. Dr. Carswell does, notwithstanding, affirm, that the blood is the sole primary seat, and that he has seen cases, where the venous blood alone was contaminated by the disease. Such instances have not fallen within my own observation; and certainly, till the statement is supported by the fullest and most accurate records of the examples themselves, it will fail to command extensive assent. * * * From all which has yet been observed and settled as true, it may, I think, be assumed, that the most frequent primary locality of cancer is not in the blood, but in the molecular structure of organized tissues or parenchymata, and that the deposit of the morbid material is dependent on perverted nutrition and secretion." 381.

*Diagnosis.*—"Simple engorgement, hypertrophy, and induration, are less hard, of more uniform surface, often unnaturally warm, and tender on pressure, whatever part may be affected; while, even in the early stages of cancer, the surface is irregular and rough, free from tenderness; and there is often a weight, coldness, and stony induration.

"In cancer, and the simpler affections already mentioned, there is a marked difference in the mucous membrane, covering the cervix. In the former it is of a dull white, or slightly grey, colour: in the latter it is much redder and more vascular, and often morbidly sensitive." 382.

*Prognosis and Duration.*—The prognosis to be delivered will depend much upon the stage at which the disease is seen. But seeing the possibility of curability in the early stages, and of prolonging life even in the latter ones, the practitioner will be cautious in not prostrating the energies of his patient by precipitately announcing the hopelessness of her case. The mean duration of cancer is not yet determined, and the period of twenty months, mentioned by Lever, is correct only if it embraces merely the second or ulcerative stage. The final termination is sometimes very sudden and unexpected, the possibility of which must be borne in mind, but not confounded with temporary failure of strength, capable of being relieved by tonic medicines.

*Prophylactic and Curative Means.*—Before entering upon these, the author states the precise condition of parts which will admit of their application.

"The muciparous glands, in the interior of the cervix, may be hard, and of the size of small shot, and pressure on them may induce pain, and yet, if the mucous membrane covering them be not ulcerated, a restoration to a healthy state may, by proper treatment, be fairly hoped for. I am aware that, in a slight degree, such a state of the muciparous glands may occasionally exist, as the result of irritation, induced by various causes, as painful and excessive intercourse, dysmenorrhœa, &c.; but the effect is then generally transient, and unaccompanied by the more permanent symptoms already mentioned. These little indurated glands are often associated with a hard and fissured state of the os, and an enlarged and hard cervix. The turgescence of the interior of the neck, and its deep flesh-colour, both within and externally, are well-marked. The uterus is usually increased in bulk, and feels altogether thicker and more solid.

"If the vagina is at all knotted and indurated; if the cervix is united to the vagina by hardened mucous membrane, and cannot be moved freely; if the uterus generally is fixed and consolidated with the neighbouring organs, or if there be abrasion, softening, or commencing ulceration; then the case wears a very unfavorable, but not entirely hopeless, aspect, and a most cautious prognosis must be given." 386.

*General Remedies.*—Complete repose on a sofa, and an entire abstraction from considerable physical or mental exertion, and from sexual intercourse—a simple unstimulating (some have been benefited by abstinence) diet, must be insisted upon. Dr. Ashwell approves of the practice of Montgomery, of bringing the system slightly under the influence of mercury, combining it with iodine and anodynes: but it is only in the earliest stages of the affection, and in the absence of any contra-indication to its use, that this drug is admissible. Although the various medicines, such as conium, belladonna, stramonium, &c., are to be rejected as possessing any specific effect on cancer, they may be employed as mere anodynes. Other medicines of a chalybeate character are often useful, and the author particularly recommends the *iodide of iron.* He has also derived much benefit from the conjoined external and internal use of the *sesqui-oxide of iron.*

"Almost any of the preparations of this invaluable remedy may be either dissolved or made into a paste with water, and topically applied. Collating the opinions of others resting on cases, with the facts observed by myself, I am convinced that mercury and iodine, aided by iron and the horizontal posture, are the best general remedies."

*Local Remedies.*—It is upon these the author chiefly relies. *Depletion* must be performed either by cupping or leeching the perineum, vulva, or cervix, or scarifying the latter—the quantity of blood taken being sufficient to relieve the state of congestion, without inducing prostration. Dr. Ashwell usually applies from three to eight leeches to the cervix, once every seven or ten days. They form an useful preparative for the iodine.

Warm *hip-baths* are of great utility, the patient remaining immersed for upwards of an hour night and morning, the access of the water to the organs being facilitated by using a perforated speculum, which she soon learns to introduce. Most patients receive great exemption of pain and procural of rest from the baths, but some find their sufferings only increased by them, when they must be discontinued. These means are only of perceptible service, after long perseverance in their employment; and we must be content if we find we can even prevent the disease from making advances, while the greatest encouragement is derived from very slight improvement. It is only after their employment we may venture upon counter-irritation, the use of iodine, nitrate of silver, &c.

" *Iodine.*—So far as my experience has gone, the external application of this drug to the cervix is sufficient to secure its beneficial effects, especially when the friction is persevered in for ten or twelve minutes. Many patients apply it by the finger, others employ a camel-hair pencil, or sponge, mounted on a slender piece of cane. The ointment I use is the following. ℞. Iodin. pur. gr. xv., Potassa hydriod. ℈ij., Ung. cetac. ℥jss. M. ft. Ung. A portion, about the size of a small nutmeg, is to be introduced into the vagina, and rubbed into the affected cervix every night. The average time in which I have seen resolution of the induration accomplished, varies from 8 to 10, to 16 or 20 weeks; this event greatly depending upon the diligence and susceptibility of the patient. It may, however, be remarked, that while there are many individuals incapable of receiving the impression of mercury, there are few on whom iodine will not exert its accustomed influence." 392.

When it causes sickness, excitement, or irritability, it may be suspended for a week or two, during which, magnesian aperients, tonics, and nutritious diet should be given.

" *Nitrate of Silver.*—I have found this caustic most useful where the mucous tissue, lining the channel of the cervix, or around the margin of the os, has been red and tender, or where there have been obvious or slight ulcerations, or a tendency to softening. The character of the mucous membrane has generally improved after three or four applications; and, in a case I am now attending, the very unhealthy surface of an indurated cervix, and its attendant and excessive leucorrhœa, have been cured by its employment. It must be repeatedly used where there is fear of ulceration, or where, from the fetor of the discharges, and the increased pain and unhealthy aspect of the surface, the disease seems likely to make rapid progress. Severe pain is not often complained of, even when the solid nitrate is rubbed over the part. Frequently, however, where the patient has suffered pain before, the nitrate has entirely removed it, and I can speak most confidently of the advantage of repeatedly obtaining a new surface from its use. The following is a sufficiently strong lotion. Arg. nit. gr. 30 ad 40, Aq. distill. ℥iv. M.

" It is scarcely necessary to observe, that the speculum must be introduced, in order to apply topical remedies with the exactness which they require." 393.

2*nd. The Advanced Stage.*—The author protests against our abandoning even these desperate cases, for we may do much to protract life and mitigate suffering. When ulceration is decidedly established, examinations are both painful and unnecessary, but prior to this they are required, or we may mistake some of the early symptoms for those attendant upon an advanced degree of the malady which really does not exist. The amount of suffering to be relieved varies in different cases, and at most we can only offer palliatives.

The *discharges* vary much in appearance, abundance, and fetor, and their temporary cessation often furnishes the patient with illusive hopes of amendment. As long as these are not excessive in quantity, mere tepid or cold-water injections suffice. At other times those of an emollient, slightly stimulant, or astringent character are required. When their prolonged use does not seem to be attended with benefit, or even produces pain, they must be discontinued, as also when they produce hæmorrhage. The nitrate of silver (gr. x. ad xx. to oz.) or the sulphate iron (ʒj. to pint) are often useful; but in the most advanced stages we are obliged to be content with mere tepid water containing a little spirit, and the chloride of lime as a corrector of the fetor. *Hæmorrhage* sooner or later comes on in almost every case, and sometimes to an extent rendering it wonderful how the patient should ever rally. The usual local means of restraining it must be had recourse to, but are often without avail. The *pain*, in some cases of even extensive ulceration, is by no means severe, while, in others, nothing can surpass its torments; and, indeed, it has sometimes terminated life at once, by inducing convulsions or other cerebral disorder. The author, although at first not rejecting the milder anodynes, relies eventually almost entirely upon *laudanum*, which disorders less than the preparations of morphia, after long use. He begins with as small a dose as possible, and suspends it when practicable. He finds its efficacy increased, and its liability to disagree diminished by adding to each teaspoonful of the tra. opii a tablespoonful of sp. lavand. c. and of brandy.

In reference to *Excision of the Cervix Uteri*, Dr. Ashwell considers, that our improved modes of examination, and knowledge of the diseases of this part, will enable the revival of the operation to be followed by greater success, than that which formerly attended it. How seldom cases will be found, to which it can be properly applied, may be judged of from the fact, that the author, so alive to the necessity of early examination in suspected cases, and so practised in conducting it, has for years been in vain seeking for a case, in which he could recommend its performance. He justly supposes that many of Lisfranc's cases of excision were performed when no cancer existed; while the testimony of his assistant, M. Pauly, proves the disgraceful falsification of the results this able, but unprincipled, surgeon was guilty of.

The *removal of the entire uterus* has been performed in cancer, both here and on the Continent; but, skilfully as the operation may have been conducted by Blundell, we hope never to hear of its having been repeated.

### Simple Ulceration of the Cervix and Os Uteri.

As there may be induration so may there be ulceration, independent of malignancy, undetected it no doubt frequently heals without any remedy, and, when left to itself does not degenerate into a malignant sore. Slight syphilitic sores, abrasion produced by excessive intercourse, instrumental delivery, abscess, &c. are not uncommon. The long-continued suffering and discharges sometimes existing *after coition* doubtless often result from this cause, but this frequently cannot for long be ascertained, owing to the delicacy which raises objections to the employment of the speculum. When inspection is permitted the appearances are various.

"1. Sometimes they are mere erosions of the mucous surface, redder than the sound membrane around, and the edges sharp and well defined. Such are not unfrequently seen after acrimonious leucorrhœa, the consequence of high living and excessive sexual indulgence. 2. The ulcers of the cervix are occasionally numerous, varying in size from a small pea to a sixpence or shilling, the larger ones being evidently formed by a coalition of the smaller; and long neglect may have induced roughness of surface and greater depth; and the colour may be a darker red. In such sores there is commonly pain on pressure, and the speculum causes bleeding. The discharge, too, may be sanguineous, and of a yellow or dirty-white colour, but usually, when there is no want of cleanliness, without fetor. 3. There is an ulceration following protracted local irritation from pessaries, sponges, and *contrivances to prevent conception*, in which the cervix is enlarged and spongy, with increased heat and great tenderness on pressure, and an open state of the os. In such cases bleeding frequently occurs.

"Every remark now made may be verified in simple ulceration, attendant on entire procidence of the womb. We all know the varying extent and depth of such ulcers, and how extremely difficult they are to heal. I believe, that partial inflammation of the cervix, resulting in simple ulceration, is by no means uncommon; an opinion which will become confirmed by the greater prevalence now than formerly, of examinations by the speculum. I am not aware that temperament has much, if any, influence; but I have rarely, if ever, seen the cervix ulcerated prior to natural or vicious sexual intercourse. Prostitutes, as might be expected, are obnoxious to it." 429.

Much unhappiness in married life, great pain in intercourse, sterility, and eventually broken health, arise from these cases not being sufficiently understood; and either the practitioner not urging, or the patient not consenting to, the indispensable inspection of the part.

If the patient be seen very early, ulceration, though not actually existing, may be imminent, and scarification, the application of leeches, or even venesection required. At all events, complete rest and hip-baths are essential, as also are mild aperients, sexual abstinence, and a spare diet. Mild injections (*e. g.* ox. zinc. ʒiij.—iv. ad ℥vi. aq.) used three or four times a day, will often cure. Mercurial and other ointments may be tried. The application of the *nitrate of silver* is highly beneficial.

### CORRODING ULCER OF THE UTERUS.

First described by Dr. John Clarke, this is a rare disease; for while we see one case of it we may see 90 or 100 of cancer. It is less painful and of slower progress. Examined by the speculum, when its diagnosis becomes easy, it much resembles lupus of external parts. There is no accompanying induration or immoveability, and defined by an accurate line of demarcation, the uterus beyond this is found healthy in structure. Nitrate of silver, if applied early, may perhaps arrest its progress in some degree.

### CAULIFLOWER EXCRESCENCE OF THE UTERUS.

This, although a commoner disease than the last, is infinitely more rare than cancer, and differs from both in the absence of pain—attention being chiefly directed to it by the abundant quantity of attendant watery discharge. The exhausting effects of this and subsequent hæmorrhages at last wear the patient out, usually in two or three years, but sometimes not until a very protracted period. The malady does not extend beyond the cervix, or invade other parts, but when removed is always reproduced. Still, life may sometimes be prolonged by the operation for several years, but how far this is the case will much depend upon the perseverance of the patient in quietude, a careful diet, the use of injections, &c.; for, in most cases, not above a year or two elapses before the fungus re-appears.

### Occlusion and Rigidity of the Cervix Uteri.

The object of this chapter is to enforce the preferability of incision over any other means of overcoming the obstacle. In reference to *occlusion*, the author says—

"That incision is the safest remedy, where the os is in a state of firm and complete closure; or, in other words, where the uterus, so far as its lower orifice is concerned, is imperforate."

In some women this orifice is very minute normally, a mere chink or small circular aperture existing; and, in such cases, a very insignificant amount of local inflammation, after conception, may produce complete occlusion. The condition of the parts, unattended with induration, may be quite natural in all other respects. In such cases very injurious delay sometimes takes place under the fallacious idea, that a mere obliquity of the os is present, to be righted by the parturient efforts, or that the occlusion, when allowed to be present, may be overcome by the efforts of Nature and depletion. Dr. Ashwell has met with no case of serious protraction caused by obliquity, and if, after some hours' active labour, no os be discovered, delay on this grouud will be unjustifiable. Fatal results have followed by delaying interference until collapse, or rupture of the uterus, have occurred. Even the prior employment of bleeding must be cautiously recommended, for, in many cases, it will only enfeeble the powers of the patient. If the practitioner has determined an occlusion of the os to exist, he must not delay giving the necessary relief; and he can accomplish this much more effectually and safely by careful *incision*, than by forcing the female catheter, or other blunt instrument, through the obstruction—impracticable indeed as he would find it to do this in many cases, and dangerous in all, by reason of the increased liability to rent and contusion of the uterus. A mere membranous obstruction would alone justify such means.

As to *Rigidity*—

"That in examples of such extreme rigidity of the os, when, after hours of strong uterine effort, the power of dilatation is entirely absent, whether such rigidity arise from disease in the structural organization of the part, or has resulted from previous laceration and ulceration, incision is the best and safest treatment; far preferable to protracted and powerful dilatation of the os by the finger; or, on the principle of non-interference, to leaving the case to the natural efforts."

Without wishing to recommend having a too precipitate recourse to the knife, the author is convinced that many fatal results are due to having delayed this too long. The precise moment when we should cease to place any reliance on bleeding, antimony, fomentations, &c. is not, certainly, so easily determined, as in the case of occlusion; but, much more danger is to be apprehended, from the too long delay, than from the too early resort to the operation. Dilatation with the finger cannot be performed with the same impunity, as where there has been no structural or local malady producing the rigidity; and the case is in no-wise analogous to that in which we dilate a healthy os, for the arrest of hæmorrhage. "Where the cervix is rigid, contracted, and diseased, and the os so small as scarcely to be recognised, powerful, and long-continued, artificial dilatation must be a dangerous remedy. It is scarcely to be expected that it should relax the parts, and lead to dilatation; it is much more likely that it should irritate, and thus induce inflammation, gangrene, and death."

Incision, even, is not always so simple a matter as it would seem at first.

"The simplest, perhaps, of the examples of rigid os uteri, is where a very contracted orifice is surrounded by a structure almost entirely undilatable. In such a case, although there may be little indication of organic change, still, if there be a total absence of the power of dilatation after the use of free venesection and antimony—time having been allowed for their beneficial effects—such a case cannot be trusted long with safety, either to the natural efforts or artificial dilatation. Other examples are not so simple as this. Many, probably the majority, are the consequence of some previous morbid occurrence. The os and cervix may have been injured in a former labour; abscesses, ulcerated surfaces, and cicatrizations may have taken place; thus, the uterine orifice may have become nearly, if not entirely, closed; and the relative situation of the bladder, urethra, and vagina, so altered, as to render the division of parts much more hazardous and difficult; or it may be, that a hard tumor, or a more malignant and active deposit, has imbedded itself in these parts, totally altering the os and the natural structure of the cervix." 452.

We have, however, no choice. Artificial dilatation is inefficient, or, if powerful, dangerous; delay is death. Incision, when performed in proper time, is safe and effective, and causes very little pain. In one of the cases detailed, the patient submitted, with good effect to the operation, in four successive pregnancies.

"The safety of incision consists in its preventing unlimited and extensive laceration. So long as division by the knife, and the subsequent tearing of the parts, is confined to the os and cervix, and does not extend beyond the reflection of the mucous surface of the vagina over these parts, recovery is almost certain; whereas, if the parts be left to rupture of themselves, the body and fundus of the uterus, and their peritoneal investment, are pretty sure to be implicated, and the result will then most probably be fatal." 468.

In concluding a careful analysis of this volume, we must express the great satisfaction we have derived from its perusal; and urge its purchase upon those who wish to be in possession of a really valuable work. Each division of the subject is illustrated by a well chosen selection of cases, which being printed in a smaller type, while they much increase the value of the work, do not proportionally augment its size or price. Works of any magnitude, are much better issued in parts, their purchase being thus rendered more convenient; but both authors and publishers should make it a point of honour, as they would find it one of profit, that these should appear punctually at the periods promised. The paging of the present work being continuous, the three parts may be bound in one volume.

